# Synthesis, Spectroscopic Characterization and Antimicrobial Potential of Certain New Isatin-Indole Molecular Hybrids

**DOI:** 10.3390/molecules22111958

**Published:** 2017-11-15

**Authors:** Reem I. Al-Wabli, Azza S. Zakaria, Mohamed I. Attia

**Affiliations:** 1 Department of Pharmaceutical Chemistry, College of Pharmacy, King Saud University, P.O. Box 2457, Riyadh 11451, Saudi Arabia; ralwabli@KSU.EDU.SA; 2 Department of Microbiology and Immunology, Faculty of Pharmacy, Alexandria University, Alexandria 21500, Egypt; azzazakaria@yahoo.com; 3 Medicinal and Pharmaceutical Chemistry Department, Pharmaceutical and Drug Industries Research Division, National Research Centre (ID: 60014618), El Bohooth Street, Dokki, Giza 12622, Egypt

**Keywords:** 5-methoxyindole, isatin, antibacterial, antifungal

## Abstract

Molecular hybridization has a wide application in medicinal chemistry to obtain new biologically active compounds. New isatin-indole molecular hybrids **5a**–**n** have been synthesized and characterized by various spectroscopic tools. The in vitro antimicrobial potential of the prepared compounds **5a**–**n** was assessed using diameter of the inhibition zone (DIZ) and minimum inhibitory concentration (MIC) assays against a panel of Gram-negative bacteria, Gram-positive bacteria and fungi. Most of the synthesized compounds **5a**–**n** showed weak activities against Gram-negative bacteria while compounds **5b** and **5c** exhibited good activities against Gram-positive bacteria. On the other hand, compound **5j** emerged as the most active compound towards *Candida albicans* (*C. albicans*), with an MIC value of 3.9 µg/mL, and compound **5g** as the most active congener towards *Asperagillus niger* (*A. niger*), with an MIC value of 15.6 µg/mL. Moreover, compound **5h** manifested the best anti-*P. notatum* effect, with an MIC value of 7.8 µg/mL, making it equipotent with compound **5g**.

## 1. Introduction

Resistance to the currently available chemotherapeutic antimicrobial agents has become an attractive subject of interest over the past 10 years. Understanding antimicrobial drug resistance mechanisms plays a crucial role in developing effective therapeutic and prophylactic strategies to preclude the problems that are encountered with resistant pathogens. The development of antimicrobial resistance microbes might occur via upregulation of genes managing drug efflux, alteration of the structure or levels of the molecular targets, or decreased affinity of the antimicrobial agents for their targets. Potential strategies to overcome antimicrobial resistance include the development of new antimicrobial candidates with better activity, combination therapy and adjunctive immune therapy with cytokines [1,2,3].

**Figure 1:**
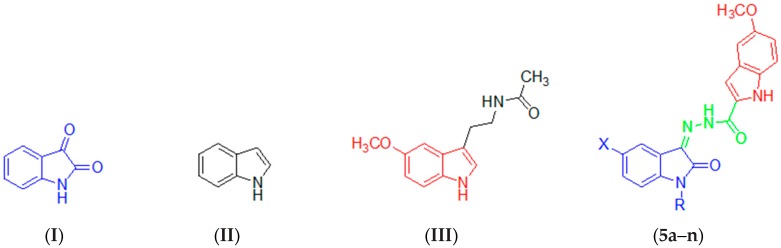
Chemical structures of isatin (**I**), indole (**II**), melatonin (**III**), and the target compounds **5a**–**n**.

Isatin (**I**, 1*H*-indole-2,3-dione; Figure 1) is a heterocyclic compound that was first synthesized nearly 170 years ago by Erdmann and Laurent [4,5]. Thereafter, it has been identified as an endogenous multifunctional compound in plants [6], fungi [7], marine organisms [8], and mammals [9]. Isatin contains a number of chemically reactive function groups that broaden its synthetic utility. Therefore, it is considered as a privileged substrate in both organic and medicinal chemistry, and it has been extensively used for the construction of diverse bioactive compounds endowed with a broad spectrum of biological activities [10,11,12,13,14,15,16,17]. Among these activities, the antiprotozoal activity exhibited certain isatin-β lactame conjugates [18]. Recently, the potent anti-*Trichomonas vaginalis* activity of an *N*-propyl tethered 5-bromo bis-isatin derivative has been reported [19].

On the other hand, indole (**II**; Figure 1) is another privileged bicyclic structure that is found in diverse molecules with broad range of pharmacological activities representing various important classes in drug discovery [20,21]. During the past decade, hundreds of published articles have reported on the isolation or synthesis of indole-bearing natural products, including complex indole alkaloids, particularly those with novel skeletons or potent bioactivities or those possessing synthetic value [22]. Among these alkaloids, indole alkaloids with the 3,3-dimethylallyl (prenyl) substituent on their scaffold constitute outstanding secondary metabolites in cyanobacteri, fungi and certain marines [23]. Indole-bearing bioactive compounds can modulate multiple receptors with a high affinity, and hence their application is to a wide range of therapeutic areas, such as analgesics [24], anti-inflammatories [25], antitumors [26], antimicrobials [27], cyclooxygenase (COX)-2 inhibitors [28], efflux pump inhibitors [29] and GABA_A_ agonists [30].

Melatonin (MLT, **III**; Figure 1) is a naturally occurring hormone incorporating 5-methoxyindole moiety, and it binds to three types of melatonin receptors to exert its diverse biological activities [31,32,33]. Moreover, examination of the literature reveals that a number of bioactive indole-bearing compounds have the 5-methoxyindole fragment in their structure [34,35,36,37].

Pharmacophore hybridization is a well-known beneficial approach in medicinal chemistry to obtain new bioactive compounds [38,39]. The bioactive molecular hybrids usually result from the hybridization of either two complementary pharmacophores or from pharmacophores with different modes of action. The combined pharmacophores may address the active site of different targets with a possible overcoming of drug resistance and exertion of synergetic effects [40]. In the present report, certain isatin-indole molecular hybrids, compounds **5a**–**n**, have been successfully synthesized to be evaluated as new antimicrobial agents. 

## 2. Results and Discussion

### 2.1. Chemistry

**Scheme 1:**
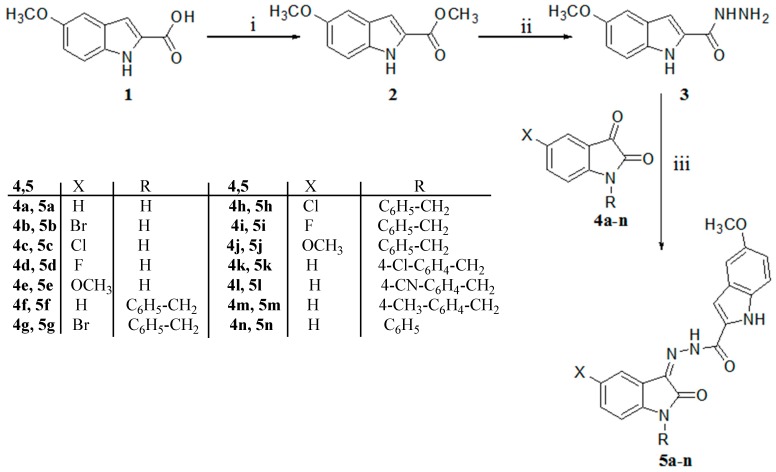
Synthetic pathway to achieve the target compounds **5a**–**n**. Reagents and conditions: (**i**) Methanol and drops of H_2_SO_4_ under reflux for 4 h; (**ii**) methanol and H_2_N–NH_2_·H_2_O under reflux for 2 h; (**iii**) absolute ethanol and 10 drops of acetic acid under reflux for 4 h.

The title compounds **5a**–**n** have been successfully achieved as portrayed in Scheme 1. The synthesis was commenced by esterification of the commercially available 5-methoxyindole-2-carboxylic acid (**1**) in methanol in the presence of a catalytic amount of sulfuric acid according to the previously reported method [34]. Subsequently, the methyl 5-methoxyindole-2-carboxylate (**2**) was allowed to react with hydrazine hydrate in methanol to afford the acid hydrazide derivative **3**. The isatins **4a**–**n** were reacted with compound **3** in the presence of a catalytic amount of glacial acetic acid to yield the title compounds **5a**–**n**. The (*Z*)-configuration of the imine double bond of the title compounds **5a**–**n** was previously confirmed via single crystal X-ray analysis of an analogous compound [41].

### 2.2. Antimicrobial Evaluation 

#### 2.2.1. Antimicrobial Susceptibility Testing 

Table 1 shows the diameter of the inhibition zone (DIZ) values after the application of the test samples **5a**–**n** and the challenging of these with various bacterial and fungal pathogens. The results indicated a significant increase in the DIZs on the application of samples **5h** and **5i** against a *Pseudomonas aeruginosa* (*Ps. aeruginosa)* isolate, as the DIZ values were 18 and 16 mm, respectively, as compared to 0 mm for the reference standard, ampicillin (AMP). Additionally, compounds **5f**, **5h** and **5i** displayed a DIZ value of 12 mm toward *Klebsiella pneumoniae (K. pneumoniae)* isolate, while the reference standard AMP was inactive. 

Against Gram-positive isolates however, clear inhibition zones were observed upon the application of most test samples, but these were weaker than those of the reference standard, AMP.

On the other hand, various test samples showed a DIZ value against different fungal isolates that was comparable to that of the reference standard, antifungal fluconazole (FLC). Compound **5j** was the most active congener toward *Candida albicans (C. albicans)*, with a DIZ value of 29 mm, while this was 21 mm for FLC. Compounds **5e**, **5k** and **5n**, however, exhibited a DIZ value of 18 mm against *Asperagillus niger (A. niger)*, as compared with 16 mm for FLC. Moreover, compounds **5e** and **5j** gave DIZ values comparable to that of FLC against *Penicillum notatum (P. notatum).*

Table 2 illustrates the MIC values of the test samples **5a**–**n** along with the reference standards, AMP for bacterial isolates and FLC for fungal isolates, against the tested pathogens. Generally, all of the tested compounds **5a**–**n** had a clear inhibitory effect on the *Bacillus subtilis (B. subtilis)* isolate, ranging from 2- to 6-fold less than AMP. Compounds **5b** and **5c** manifested a clear anti-Gram-positive activity that was about 4–5-fold more potent than AMP, except against *Enterococcus fecalis (E. fecalis)*. Although compound **5i** showed almost no activity against the tested Gram-positive isolates, except for the *B. subtilis* isolate, it showed better activity than AMP against *Ps. aeruginosa* with an MIC value of 62.5 µg/mL as compared with AMP (MIC value of 1000 µg/mL).

On the other hand, compound **5g** displayed a potent antifungal activity against the tested yeast and the filamentous fungi, as its MIC value was half that of FLC against *C. albicans* and *A. niger*. Moreover, compounds **5g** and **5h** were equipotent toward *P. notatum* with an MIC value of 7.8 µg/mL, making them approximately 32 times more potent than FLC (MIC value of 250 µg/mL). Compound **5j** was the most active anti-*C*. *albicans* candidate with an MIC value of 3.9 µg/mL, making it about 4 times more potent than FLC (MIC value of 15.6 µg/mL).

#### 2.2.2. Scanning Electron Microscopy

**Figure 2:**
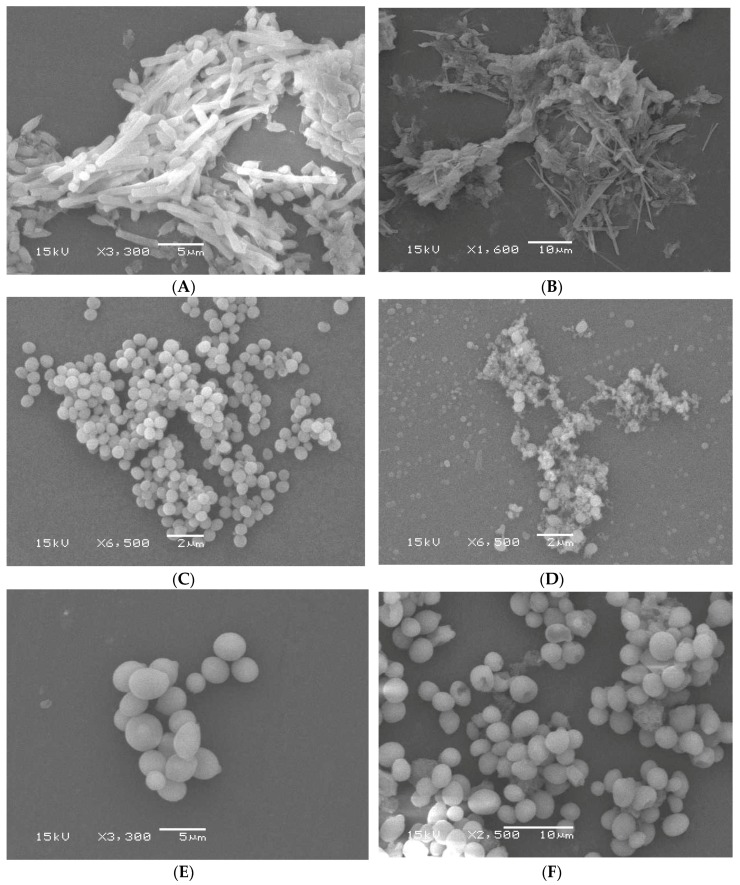
(**A**) Scanning electron microscopy (SEM) image of normal *Bacillus subtilis* (*B. subtilis*) cells; (**B**) SEM of compound **5b** after 24 h incubation with *B. subtilis*; (**C**) SEM of normal *Staphylococcus aureus (S. aureus)* cells; (**D**) SEM of compound **5b** after 24 h incubation with *S. aureus*; (**E**) normal mount of *Candida albicans (C. albicans)* cells; (**F**) SEM of compound **5j** after 24 h incubation with *C. albicans*.

Bacterial and fungal cells were photographed using electron microscopy to compare morphological alterations after the addition of compounds **5b** and/or **5j** to the pathogens with subsequent incubation for 24 h (Figure 2). For each isolate, the most illustrative photograph was chosen even if morphologically normal organisms were still observed in the mount.

The scanning electron microscopy (SEM) appearance of untreated *B. subtilis* cells is shown in Figure 2A.

The bacteria appeared as a long, thick bacilli chain-like structure, and even some oval spores were clear in the mount. However, after the application of compound **5b**, the cells were totally deformed, having a spindle distorted shape with aggregation and exhibiting fusion of the whole cells together (Figure 2B). Figure 2C,D show the *Staphylococcus aureus (S. aureus)* isolate before and after treatment, respectively. The bacteria were roughly spherical and smooth with a clear grape-like arrangement. Compound **5b** exhibited a profound alteration of the morphological structure of the cells and clumping of the cocci cells. Moreover, bacterial lysis was observed for most of the cells (Figure 2D).

On the other hand, upon the application of compound **5j** on *C. albicans* cells, the obtained results were a little different. The normal mount of *C. albicans* cells under SEM showed clear oval cells having a scattered arrangement, in addition to few budding cells (Figure 2E). However, after adding compound **5j**, no clumping of the fungal cells was shown, but abnormal forms were visible with a clear change in their oval morphology (Figure 2F). Some cells were destructed and fused, while others shrunk with clear pores on their outer surface, which resulted in the loss of their normal oval morphology (Figure 2F).

## 3. Experimental

### 3.1. General

The melting points were measured using a Gallenkamp melting point device and are uncorrected. The NMR samples of the synthesized compounds **5a**–**n** were dissolved in dimethyl sulfoxide (DMSO)-*d*_6_, and the NMR spectra were recorded using a Bruker NMR spectrometer (Bruker, Reinstetten, Germany) at 500 MHz for ^1^H and 125.76 MHz for ^13^C at the Research Center, College of Pharmacy, King Saud University, Saudi Arabia. Chemical shifts are expressed in δ-values (ppm) relative to TMS as an internal standard. Elemental analyses were carried out at the Microanalysis Laboratory, Cairo University, Cairo, Egypt, and the results agreed favorably with the proposed structures within ±0.4% of the theoretical values. Mass spectra were recorded using Agilent Quadrupole 6120 LC/MS with an electrospray ionization (ESI) source (Agilent Technologies, Palo Alto, CA, USA). Compounds **4a**–**e** and **4n** are commercially available.

### 3.2. Chemistry

#### 3.2.1. Synthesis of Methyl 5-Methoxy-1*H*-Indole-2-Carboxylate (**2**)

5-Methoxy-1*H*-indole-2-carboxylic acid (**2**) was subjected to esterification in methanol and a catalytic amount of sulfuric acid according to the reported method [42]. Its spectral data were in accordance with those in the literature [43].

#### 3.2.2. Synthesis of 5-Methoxy-1*H*-Indole-2-Carbohydrazide (**3**)

Compound **2** (5 mmol) was suspended in methanol, and hydrazine hydrate (50 mmol) was added. The reaction mixture was refluxed for 3 h under stirring and was then cooled to ambient temperature. The precipitated solid was filtered and dried to afford compound **3** [44] in 90% yield as a white solid (m.p. 266–268 °C), which was pure enough to be used for further reactions. ^1^H-NMR (DMSO-*d*_6_) ppm: 3.76 (s, 3H, OCH_3_), 4.52 (s, 2H, NH_2_), 6.84 (dd, *J* = 2.5, 8.5 Hz, 1H, H_ar._), 7.04 (d, *J* = 1.0 Hz, 1H, H_ar._), 7.07 (d, *J* = 2.5 Hz, 1H, H_ar._), 7.36 (d, *J* = 8.5 Hz, 1H, H_ar._), 9.75 (s, 1H, NH), 11.46 (s, 1H, NH); ^13^C-NMR (DMSO-*d*_6_) ppm: 55.2 (OCH_3_), 101.7, 101.9, 113.0, 114.2, 127.2, 130.8, 131.6, 153.7 (C_ar._, CH_ar._), 161.2 (C=O); MS *m*/*z*: 206 [M + 1]^+^, 228 [M + 23]^+^.

#### 3.2.3. General Procedure of the Synthesis of the *N*-Benzylated isatins **4f**–**m**

Benzyl bromide/chloride (10 mmol) was added to a stirred suspension containing the appropriate isatin (10 mmol) and potassium carbonate (2.76 g, 20 mmol) in dimethylformamide (10 mL). The reaction mixture was stirred at room temperature for 18 h, poured onto ice-cold water, filtered and dried to give the respective *N*-benzylated isatins **4f**–**m**.

1-Benzyl-1*H*-indole-2,3-dione (**4f**): Orange powder; m.p. 138–140 °C [45].
1-Benzyl-5-bromo-1*H*-indole-2,3-dione (**4g**): Orange powder; m.p. 148–150 °C [46].
1-Benzyl-5-chloro-1*H*-indole-2,3-dione (**4h**): Orange powder; m.p. 138–140 °C [47].
1-Benzyl-5-fluoro-1*H*-indole-2,3-dione (**4i**): Light-red powder; m.p. 135–137 °C [48].
1-Benzyl-5-methoxy-1*H*-indole-2,3-dione (**4j**): Light brown powder; m.p. 123–125 °C [49].
1-(4-Chlorobenzyl)-1*H*-indole-2,3-dione (**4k**): Orange powder; m.p. 168–170 °C [50].
4-[(2,3-Dioxo-2,3-dihydro-1*H*-indol-1-yl)methyl]benzonitrile (**4l**): Orange powder; m.p. 217–219 °C [50].
1-(4-Methylbenzyl)-1*H*-indole-2,3-dione (**4m**): Orange powder; m.p. 143–145 °C [51].

#### 3.2.4. General Procedure for the Synthesis of the Target Compounds **5a**–**n**

The appropriate isatin derivative (**4a**–**n**, 5 mmol) was added to a stirred suspension containing the carbohydrazide derivative **3** (5 mmol) and a catalytic amount of glacial acetic acid in absolute ethanol (10 mL). The reaction mixture was stirred under reflux for 4 h and the produced solid was filtered while hot. The collected solid was washed with warm ethanol and dried to afford the title compounds **5a**–**n** in moderate to good yields. Analytical samples of compounds **5a**–**n** were obtained via their re-crystallization from a dimthylformamide/ethanol (1:3) mixture.

*5-Methoxy-N’-[(3Z)-2-oxo-1,2-dihydro-3H-indol-3-ylidene]-1H-indole-2-carbohydrazide* (**5a**): Yellow powder; m.p. > 300 °C (yield 80%); IR (KBr, ν cm^−1^): 3361 (NH) and 1691, 1656 (2 × C=O); ^1^H-NMR (DMSO-*d*_6_) ppm: 3.79 (s, 3H, OCH_3_), 7.12–7.14 (m, 2H, H_ar._), 7.18 (d, *J* = 2.0 Hz, 1H, H_ar._), 7.39 (s, 1H, H_ar._), 7.41 (s, 1H, H_ar._), 7.42–7.44 (m, 1H, H_ar._), 7.52 (s, 1H, H_ar._), 8.04 (d, *J* = 8.0 Hz, 1H, H_ar._), 10.89 (s, 1H, NH), 11.64 (s, 1H, NH), 11.85 (s, 2H, NH); ^13^C-NMR (DMSO-*d*_6_) ppm: 55.2 (OCH_3_), 102.1, 110.7, 113.4, 113.5, 115.6, 116.2, 120.9, 121.8, 122.7, 126.6, 127.3, 132.5, 132.7, 143.9, 154.1 (C_ar._, CH_ar._, C=N), 161.8, 164.8 (2 × C=O); MS *m*/*z*: 335 [M + H]^+^, 357 [M + Na]^+^.

*N’-[(3Z)-5-Bromo-2-oxo-1,2-dihydro-3H-indol-3-ylidene]-5-methoxy-1H-indole-2-carbohydrazide* (**5b**): Yellow powder; m.p. > 300 °C (yield 47%); IR (KBr, ν cm^−1^): 3356 (NH) and 1695, 1654 (2 × C=O); ^1^H-NMR (DMSO-*d*_6_) ppm: 3.79 (s, 3H, OCH_3_), 7.14 (d, *J* = 8.5 Hz, 2H, H_ar._), 7.19 (s, 1H, H_ar._), 7.44 (d, *J* = 8.5 Hz, 1H, H_ar._), 7.45 (d, *J* = 8.0 Hz, 1H, H_ar._), 7.57 (s, 1H, H_ar._), 8.20 (s, 1H, H_ar._), 11.00 (s, 1H, NH), 11.49 (s, 1H, NH), 11.82 (s, 2H, NH); ^13^C-NMR (DMSO-*d*_6_) ppm: 57.7 (OCH_3_), 102.6, 108.2, 112.4, 113.9, 116.9, 117.1, 122.1, 126.2, 126.6, 127.8, 129.2, 132.4, 132.9, 143.1, 154.5 (C_ar._ and CH_ar._, C=N), 162.1, 165.1 (2 × C=O); MS *m*/*z*: 413 [M]^+^.

*N’-[(3Z)-5-Chloro-2-oxo-1,2-dihydro-3H-indol-3-ylidene]-5-methoxy-1H-indole-2-carbohydrazide* (**5c**): Yellow powder; m.p. > 300 °C (yield 65%); IR (KBr, ν cm^−1^): 3360 (NH) and 1691, 1654 (2 × C=O); ^1^H-NMR (DMSO-*d*_6_) ppm: 3.79 (s, 3H, OCH_3_), 6.94–6.99 (m, 2H, H_ar._), 7.23 (s, 1H, H_ar._), 7.34–7.47 (m, 2H, H_ar._), 7.57 (s, 1H, H_ar._), 8.20 (s, 1H, H_ar._), 11.00 (s, 1H, NH), 11.83 (br. s, 2H, NH); ^13^C-NMR (DMSO-*d*_6_) ppm: 57.7 (OCH_3_), 102.6, 108.3, 112.4, 113.3, 113.4, 116.5, 117.1, 126.3, 127.3, 128.7, 132.5, 133.7, 134.7, 142.9, 153.9 (C_ar._, CH_ar._, C=N), 161.4, 164.5 (2 × C=O); MS *m*/*z*: 369 [M]^+^.

*N’-[(3Z)-5-Fluoro-2-oxo-1,2-dihydro-3H-indol-3-ylidene]-5-methoxy-1H-indole-2-carbohydrazide* (**5d**): Yellow powder; m.p. > 300 °C (yield 61%); IR (KBr, ν cm^−1^): 3371 (NH) and 1695, 1653 (2 × C=O); ^1^H-NMR (DMSO-*d*_6_) ppm: 3.79 (s, 3H, OCH_3_), 6.92–6.96 (m, 2H, H_ar._), 7.19 (d, *J* = 2.5 Hz, 1H, H_ar._), 7.28 (ddd, *J* = 2.5, 9.0, 9.0 Hz, 1H, H_ar._), 7.40 (dd, *J* = 2.5, 9.0 Hz, 1H, H_ar._), 7.58 (s, 1H, H_ar._), 8.01 (d, *J* = 8.5 Hz, 2H, H_ar._), 10.89 (s, 1H, NH), 11.75 (s, 1H, NH), 11.83 (s, 1H, NH); ^13^C-NMR (DMSO-*d*_6_) ppm: 55.2 (OCH_3_), 102.1, 113.4, 113.5, 116.4, 127.3, 128.7, 132.5, 138.5, 140.2, 153.9 (C_ar._, CH_ar._, C=N), 163.2, 164.9 (2 × C=O), 111.3 (C_3__’__-F_, *J* = 7.6 Hz, C_ar._), 113.7 (C_2__’__-F_, *J* = 26.3 Hz, C_ar._), 115.8 (C_3__’__-F_, *J* = 9.0 Hz, C_ar._), 118.8 (C_2__’__-F_, *J* = 23.8 Hz, C_ar._), 157.4 (C_1__’__-F_, *J* = 234.0 Hz, C_ar._); MS *m*/*z*: 353 [M + H]^+^, 375 [M + Na]^+^.

*5-Methoxy-N’-[(3Z)-5-methoxy-2-oxo-1,2-dihydro-3H-indol-3-ylidene]-1H-indole-2-carbohydrazide* (**5e**): Orange powder; m.p. > 300 °C (yield 94%); IR (KBr, ν cm^−1^): 3348 (NH) and 1718, 1654 (2 × C=O); ^1^H-NMR (DMSO-*d*_6_) ppm: 3.79 (s, 3H, OCH_3_), 3.82 (s, 3H, OCH_3_), 6.86 (d, *J* = 8.5 Hz, 1H, H_ar._), 6.94 (dd, *J* = 2.5, 9.0 Hz, 1H, H_ar._), 7.01 (dd, *J* = 2.5, 8.5 Hz, 1H, H_ar._), 7.18 (d, *J* = 2.0 Hz, 1H, H_ar._), 7.39 (d, *J* = 9.0 Hz, 1H, H_ar._), 7.54 (s, 1H, H_ar._), 7.69 (s, 1H, H_ar._), 10.68 (s, 1H, NH), 11.76 (s, 1H, NH), 11.82 (s, 2H, NH); ^13^C-NMR (DMSO-*d*_6_) ppm: 55.7 (OCH_3_), 56.3 (OCH_3_), 102.6, 111.6, 112.5, 113.2, 113.8, 113.9, 116.4, 116.8, 118.6, 127.7, 127.8, 132.9, 138.0, 154.5, 154.9 (C_ar._, CH_ar._, C=N), 163.8, 165.7 (2 × C=O); MS *m*/*z*: 363 [M − H]^−^.

*N’-[(3Z)-1-Benzyl-2-oxo-1,2-dihydro-3H-indol-3-ylidene]-5-methoxy-1H-indole-2-carbohydrazide* (**5f**): Yellow powder; m.p. 266–268 °C (yield 69%); IR (KBr, ν cm^−1^): 3338 (NH) and 1718, 1654 (2 × C=O); ^1^H-NMR (DMSO-*d*_6_) ppm: 3.79 (s, 3H, OCH_3_), 5.02 (s, 2H, CH_2_), 6.95 (d, *J* = 7.5 Hz, 1H, H_ar._), 7.05 (d, *J* = 7.5 Hz, 1H, H_ar._), 7.16–7.19 (m, 2H, H_ar._), 6.28–7.30 (m, 1H, H_ar._), 7.36–7.37 (m, 2H, H_ar._), 7.39–7.45 (m, 4H, H_ar._), 7.56 (s, 1H, H_ar._), 8.11 (d, *J* = 7.5 Hz, 1H, H_ar._), 11.75 (s, 1H, NH), 11.87 (s, 1H, NH); ^13^C-NMR (DMSO-*d*_6_) ppm: 43.2 (CH_2_), 57.7 (OCH_3_), 102.6, 110.4, 113.9, 115.7, 116.8, 122.9, 126.9, 127.6, 127.8, 127.9, 129.1, 129.2, 129.4, 132.9, 133.1, 133.5, 136.7, 144.3, 154.5 (C_ar._, CH_ar._, C=N), 163.4, 164.1 (2 × C=O); MS *m*/*z*: 425. [M + H]^+^, 447 [M + Na]^+^. 

*N’-[(3Z)-1-Benzyl-5-bromo-2-oxo-1,2-dihydro-3H-indol-3-ylidene]-5-methoxy-1H-indole-2-carbohydrazide* (**5g**): Yellow powder; m.p. 262–264 °C (yield 63%); IR (KBr, ν cm^−1^): 3313 (NH) and 1722, 1662 (2 × C=O); ^1^H-NMR (DMSO-*d*_6_) ppm: 3.79 (s, 3H, OCH_3_), 5.02 (s, 2H, CH_2_), 6.94–7.05 (m, 3H, H_ar._), 7.19–7.32 (m, 2H, H_ar._), 7.36–7.43 (m, 4H, H_ar._), 7.60 (d, *J* = 9.0 Hz, 1H, H_ar._), 7.82 (s, 1H, H_ar._), 8.42 (s 1H, H_ar._), 11.84 (s, 1H, NH), 11.94 (s, 1H, NH); ^13^C-NMR (DMSO-*d*_6_) ppm: 43.3 (CH_2_), 55.7 (OCH_3_), 102.6, 112.2, 113.9, 114.7, 115.8, 117.2, 127.7, 127.8, 127.9, 128.0, 129.1, 129.2, 133.0, 134.0, 135.3, 135.8, 136.4, 142.0, 154.5 (C_ar._, CH_ar._, C=N), 164.4, 164.9 (2 × C=O); MS *m*/*z*: 503 [M]^+^.

*N’-[(3Z)-1-Benzyl-5-chloro-2-oxo-1,2-dihydro-3H-indol-3-ylidene]-5-methoxy-1H-indole-2-carbohydrazide* (**5h**): Yellow powder; m.p. 256–258 °C (yield 79%); IR (KBr, ν cm^−1^): 3315 (NH) and 1726, 1649 (2 × C=O); ^1^H-NMR (DMSO-*d*_6_) ppm: 3.79 (s, 3H, OCH_3_), 5.02 (s, 2H, CH_2_), 6.94–7.12 (m, 3H, H_ar._), 7.19–7.31 (m, 2H, H_ar._), 7.32–7.48 (m, 4H, H_ar._), 7.61–7.71 (m, 2H, H_ar._), 8.31 (s, 1H, H_ar._), 11.83 (br. s, 2H, 2 × NH); ^13^C-NMR (DMSO-*d*_6_) ppm: 43.4 (CH_2_), 55.7 (OCH_3_), 102.8, 112.5, 113.1, 113.9, 114.7, 116.9, 127.7, 127.8, 127.9, 128.0, 129.1, 129.2, 133.0, 135.7, 135.9, 136.2, 136.5, 141.7, 154.3 (C_ar._, CH_ar._, C=N), 158.3, 161.7 (2 × C=O); MS *m*/*z*: 459 [M + H]^+^, 481 [M + Na]^+^.

*N’-[(3Z)-1-Benzyl-5-fluoro-2-oxo-1,2-dihydro-3H-indol-3-ylidene]-5-methoxy-1H-indole-2-carbohydrazide* (**5i**): Yellow powder; m.p. 236–238 °C (yield 93%); IR (KBr, ν cm^−1^): 3329 (NH) and 1728, 1653 (2 × C=O); ^1^H-NMR (DMSO-*d*_6_) ppm: 3.79 (s, 3H, OCH_3_), 5.02 (s, 2H, CH_2_), 6.95 (dd, *J* = 2.5, 9.0 Hz, 1H, H_ar._), 7.03–7.06 (m, 2H, H_ar._), 7.19 (d, *J* = 1.5 Hz, 1H, H_ar._), 7.24–7.35 (m, 2H, H_ar._), 7.36–7.44 (m, 4H, H_ar._), 7.62 (s, 1H, H_ar._), 8.12 (d, *J* = 8.5 Hz, 1H, H_ar._), 11.85 (br. s, 2H, 2 × NH); ^13^C-NMR (DMSO-*d*_6_) ppm: 43.4 (CH_2_), 55.7 (OCH_3_), 102.8, 111.2, 112.1, 113.8, 113.9, 116.1, 116.9, 117.2, 127.7, 127.8, 127.9, 128.0, 129.1, 129.2, 133.1, 135.9, 136.5, 140.6, 154.3 (C_ar._, CH_ar._, C=N), 157.3, 164.0 (2 × C=O); MS *m*/*z*: 443 [M + H]^+^, 465 [M + Na]^+^.

*N’-[(3Z)-1-Benzyl-5-methoxy-2-oxo-1,2-dihydro-3H-indol-3-ylidene]-5-methoxy-1H-indole-2-carbohydrazide* (**5j**): Orange powder; m.p. 248–250 °C (yield 84%); IR (KBr, ν cm^−1^): 3346 (NH) and 1705, 1668 (2 × C=O); ^1^H-NMR (DMSO-*d*_6_) ppm: 3.79 (s, 3H, OCH_3_), 3.82 (s, 3H, OCH_3_), 4.99 (s, 2H, CH_2_), 6.94–6.97 (m, 2H, H_ar._), 7.19 (d, *J* = 2.5 Hz, 1H, H_ar._), 7.26–7.31 (m, 2H, H_ar._), 7.36–7.37 (m, 4H, H_ar._), 7.40 (d, *J* = 9.0 Hz, 1H, H_ar._), 7.58 (s, 1H, H_ar._), 7.77 (s, 1H, H_ar._), 11.84 (br. s, 2H, 2 × NH); ^13^C-NMR (DMSO-*d*_6_) ppm: 43.1 (CH_2_), 55.7 (OCH_3_), 56.4 (OCH_3_), 102.8, 110.9, 113.4, 113.9, 114.9, 116.9, 117.9, 127.7, 127.8, 127.9, 129.2, 132.9, 134.1, 135.5, 136.8, 138.6, 142.6, 154.5, 155.5 (C_ar._, CH_ar._, C=N), 158.3, 161.7 (2 × C=O); MS *m*/*z*: 453 [M − H]^−^.

*N’-[(3Z)-1-(4-Chlorobenzyl)-2-oxo-1,2-dihydro-3H-indol-3-ylidene]-5-methoxy-1H-indole-2-carbohydrazide* (**5k**): Yellow powder; m.p. 273–275 °C (yield 72%); IR (KBr, ν cm^−1^): 3327 (NH) and 1724, 1654 (2 × C=O); ^1^H-NMR (DMSO-*d*_6_) ppm: 3.79 (s, 3H, OCH_3_), 5.02 (s, 2H, CH_2_), 6.94–6.97 (m, 1H, H_ar._), 7.06–7.09 (m, 1H, H_ar._), 7.16–7.21 (m, 2H, H_ar._), 7.23 (d, *J* = 2.5 Hz, 1H, H_ar._), 7.39–7.42 (m, 4H, H_ar._), 7.45–7.49 (m, 2H, H_ar._), 7.54 (s, 1H, H_ar._), 7.72 (d, *J* = 7.5 Hz, 1H, H_ar._), 8.13 (d, *J* = 7.5 Hz, 1H, H_ar._), 11.76 (s, 1H, NH), 11.87 (s, 1H, NH); ^13^C-NMR (DMSO-*d*_6_) ppm: 42.7 (CH_2_), 55.7 (OCH_3_), 102.6, 110.4, 113.8, 113.9, 115.7, 116.8, 119.9, 123.1, 127.8,129.2, 129.7, 129.9, 132.6, 132.8, 133.1, 135.2, 135.7, 142.9, 154.5 (C_ar._, CH_ar._, C=N), 161.8, 164.1 (2 × C=O); MS *m*/*z*: 457 [M − H]^−^.

*N’-[(3Z)-1-(4-Cyanobenzyl)-2-oxo-1,2-dihydro-3H-indol-3-ylidene]-5-methoxy-1H-indole-2-carbohydrazide* (**5l**): Yellow powder; m.p. 297–299 °C (yield 92%); IR (KBr, ν cm^−1^): 3344 (NH) and 1720, 1660 (2 × C=O); ^1^H-NMR (DMSO-*d*_6_) ppm: 3.79 (s, 3H, OCH_3_), 5.13 (s, 2H, CH_2_), 6.94–6.97 (m, 1H, H_ar._), 7.04–7.07 (m, 1H, H_ar._), 7.18–7.22 (m, 1H, H_ar._), 7.23 (d, *J* = 2.5 Hz, 1H, H_ar._), 7.39–7.45 (m, 1H, H_ar._), 7.56 (d, *J* = 8.5 Hz, 2H, H_ar._), 7.64 (d, *J* = 8.5 Hz, 2H, H_ar._), 7.84 (d, *J* = 2.5 Hz, 1H, H_ar._), 7.85 (d, *J* = 2.5 Hz, 1H, H_ar._), 8.16 (d, *J* = 8.0 Hz, 1H, H_ar._), 11.77 (s, 1H, NH), 11.86 (s, 1H, NH); ^13^C-NMR (DMSO-*d*_6_) ppm: 42.9 (CH_2_), 55.7 (OCH_3_), 102.7, 110.8, 113.8, 113.9, 115.8, 117.0, 119.1, 120.0, 121.3, 123.2, 127.8, 128.6, 128.8, 131.9, 133.1, 133.2, 141.9, 142.5, 142.8, 154.5 (C_ar._, CH_ar._, C=N, CN), 157.9, 161.9 (2 × C=O); MS *m*/*z*: 448 [M − H]^−^.

*5-Methoxy-N’-[(3Z)-1-(4-methylbenzyl)-2-oxo-1,2-dihydro-3H-indol-3-ylidene]-1H-indole-2-carbohydrazide* (**5m**): Yellow powder; m.p. 218–220 °C (yield 80%); IR (KBr, ν cm^−1^): 3348 (NH) and 1718, 1662 (2 × C=O); ^1^H-NMR (DMSO-*d*_6_) ppm: 2.27 (s, 3H, CH_3_), 3.79 (s, 3H, OCH_3_), 4.97 (s, 2H, CH_2_), 6.94–6.96 (m, 1H, H_ar._), 7.04 (d, *J* = 7.5 Hz, 1H, H_ar._), 7.15–7.19 (m, 4H, H_ar._), 7.26 (d, *J* = 8.0 Hz, 2H, H_ar._), 7.40 (d, *J* = 8.0 Hz, 2H, H_ar._), 7.56 (s, 1H, H_ar._), 8.11 (d, *J* = 7.5 Hz, 1H, H_ar._), 11.75 (s, 1H, NH), 11.87 (s, 1H, NH); ^13^C-NMR (DMSO-*d*_6_) ppm: 21.3 (CH_3_), 42.9 (CH_2_), 55.7 (OCH_3_), 102.6, 110.4, 111.0, 113.4, 113.9, 115.4, 115.7, 121.2, 122.9, 126.9, 127.7, 127.8, 127.9, 129.8, 133.1, 133.6, 137.2, 144.3, 154.5 (C_ar._, CH_ar._, C=N), 163.8, 164.1 (2 × C=O); MS *m*/*z*: 437 [M − H]^−^.

*5-Methoxy-N’-[(3Z)-2-oxo-1-phenyl-1,2-dihydro-3H-indol-3-ylidene]-1H-indole-2-carbohydrazide* (**5n**): Yellow powder; m.p. > 300 °C (yield 85%); IR (KBr, ν cm^−1^): 3327 (NH) and 1728, 1660 (2 × C=O); ^1^H-NMR (DMSO-*d*_6_) ppm: 3.77 (s, 3H, OCH_3_), 6.88 (d, *J* = 8.0 Hz, 1H, H_ar._), 6.96 (d, *J* = 2.0 Hz, 1H, H_ar._), 7.19 (d, *J* = 2.0 Hz, 1H, H_ar._), 7.27 (d, *J* = 7.5 Hz, 1H, H_ar._), 7.40–7.43 (m, 2H, H_ar._), 7.52–7.55 (m, 2H, H_ar._), 7.58–7.65 (m, 3H, H_ar._), 7.80 (d, *J* = 7.5 Hz, 1H, H_ar._), 8.21 (d, *J* = 7.5 Hz, 1H, H_ar._), 11.84 (s, 1H, NH), 11.87 (s, 1H, NH); ^13^C-NMR (DMSO-*d*_6_) ppm: 57.7 (OCH_3_), 102.8, 110.7, 113.9, 115.7, 116.8, 119.9, 121.4, 123.4, 124.2, 127.2, 127.4, 127.9, 129.2, 130.1, 131.9, 133.3, 134.3, 143.9, 154.6 (C_ar._, CH_ar._, C=N), 161.2, 163.4 (2 × C=O); MS *m*/*z*: 409 [M − H]^−^.

### 3.3. Antimicrobial Activity

#### 3.3.1. Antimicrobial Agents

Stock solutions (1000 μg/mL) of AMP (Sigma-Aldrich Co., St. Louis, MO, USA) and FLC (Shouguang-Fukang Pharmaceutical Ltd., Shouguang, Shandong, China) were used as a positive control for bacteria and fungi, respectively. AMP was dissolved in water, while the test compounds as well as FLC were prepared in 100% dimethyl sulfoxide (DMSO) and were diluted with sterile distilled water. The antimicrobial discs (containing 25 μg of FLC or 10 μg of AMP) were purchased from ROSCO (Neo-Sensitabs, Taastrup, Denmark) and were stored at −80 °C until use.

#### 3.3.2. Media

The bacteria were slanted on Nutrient agar (Difco Laboratories, Detroit, MI, USA), yeast was slanted on Sabouraud dextrose agar (SDA; Difco Laboratories, Detroit, MI, USA), and the fungi were slanted on potato dextrose agar (PDA; Eiken Chemical Co. Ltd., Tokyo, Japan). Muller Hinton broth (MHB) and Muller Hinton agar (MHA) were purchased from Sigma-Aldrich Co. (St. Louis, MO, USA) and were used following the manufacturer’s instructions for the antimicrobial assay. Liquid RPMI 1640 medium supplemented with L-glutamine was purchased from Sigma-Aldrich Co. (St. Louis, MO, USA), added to 2% sodium bicarbonate and 0.165 M 3-(*N*-morpholino)propanesulfonic acid (MOPS) from Dojindo Laboratories (Kumamoto, Japan), adjusted to pH 7.0, and used for the assay of the yeast and moulds. MacConkey’s agar, mannitol salt agar, cetrimide agar, and brain heart infusion broth (BHI) were obtained from Difco Laboratories (Detroit, MI, USA).

#### 3.3.3. Isolates

The following common pathogenic microorganisms were selected: Five Gram-negative organisms, namely, *Ps. aeruginosa*, *Escherichia coli* (*E. coli*), *K. pneumoniae*, *Proteus vulgaris* (*P. vulgaris*), and *Salmonella enteridis* (*S. enteridis*); four Gram-positive isolates, namely, *E. fecalis*, *S. aureus*, *B. subtilis*, and methicillin-resistant *Staphylococcus aureus* (MRSA); three fungal isolates; two mould isolates, *P. notatum* and *A. niger*; and *C. albicans*. All the isolates were obtained from King Khaled Hospital, Riyadh, Saudi Arabia.

#### 3.3.4. Culture Conditions

All clinical samples were first inoculated onto sheep blood agar (SPML Co. Ltd., Riyadh, Saudi Arabia). The plates were incubated at 37 °C for 24–48 h. The identification of isolates was performed according to the standard methods described elsewhere [52] and by the Clinical Laboratory Standards Institute [53]. Isolates were stored in BHI containing 16% (*w*/*v*) glycerol at −80 °C until further use.

#### 3.3.5. Growth of the Tested Microorganisms

Staphylococcal isolates were re-inoculated onto mannitol salt agar, and then the plates were incubated at 37 °C for 24–48 h. Mannitol fermentation was observed and recorded. Gram-negative isolates were re-inoculated onto MacConkey’s agar, and then the plates were incubated at 37 °C for 24–48 h. Lactose fermentation was observed and recorded. *Ps. aeruginosa* strains were further re-inoculated on cetrimide agar at 37 °C for 24 h.

#### 3.3.6. Determination of Minimum Inhibitory Concentrations

The MICs of AMP and/or the synthesized compounds **5a**–**n** against the bacterial isolates were determined with a microdilution test, according to the reference method of the CLSI [54]. A stock solution of pure AMP drug was prepared in sterile distilled water, while a stock solution of each of the samples under testing was prepared in DMSO to reach an initial concentration of 1000 µg/mL. The preparation of inocula for broth microdilution testing was performed in accordance with CLSI standard procedures [55], and the MIC was defined as the lowest concentration of the antibiotic or the test sample that prevented bacterial growth. The preparation of fungal inocula for the broth microdilution testing was performed in accordance with CLSI documents M27-A3 [56] and M38-A2 [57] with RPMI 1640 medium buffered to pH 7.0 and a 0.165 M MOPS buffer for all organisms. In the case of yeasts, the MICs were recorded as the lowest concentration at which a 50% decrease in turbidity relative to the turbidity of the growth control was observed. In the case of the filamentous fungi, the MICs of the test samples and FLC were recorded as the lowest concentrations at which a prominent decrease in turbidity was observed.

#### 3.3.7. Disk Diffusion Assay

The antibacterial and antifungal screenings were conducted by the disk diffusion agar methods as described previously [58]. Bacterial and fungal suspensions were adjusted to a 0.5 McFarland standard corresponding to 5 × 10^6^ CFU/mL. A 100 μL aliquot of each isolate suspension was uniformly spread onto MHA and SDA plates for the bacteria and fungi, respectively. All the test samples were dissolved in DMSO at a 1000 μg/mL concentration; AMP (10 µg) was used as a positive control for bacteria and FLC (25 µg) was used as a positive control for fungi. Sterilized paper discs with only DMSO were used as negative controls for both the bacteria and fungi. The plates were incubated under aerobic conditions at 35 °C for 24 and 48 h for the bacteria and fungi, respectively. To obtain comparable results, all prepared solutions were treated under the same conditions. The experiments were carried out in triplicate. The plates were examined for evidence of antimicrobial activities, represented by a zone of inhibition of the microorganism’s growth around the discs, and diameters of clear zones were expressed in millimeters (mm) [59].

#### 3.3.8. Scanning Electron Microscopy

Overnight *S. aureus*, *B. subtilis* and *C. albicans* cultures were diluted to obtain 10^7^ CFU/mL. The prepared suspensions were then cultured for 24 h in MHB containing 32 µg/mL of compound **5b** for the bacterial isolates and 7.8 µg/mL of compound **5j** for the *Candida* isolate (2 times their MIC values). Then these cells were prepared to be photographed before and after treatment by SEM following the procedure described elsewhere [60].

## 4. Conclusions

A new series of isatin-indole molecular hybrids **5a**–**n** has been successfully achieved. The assigned chemical structures of compounds **5a**–**n** have been confirmed by different spectroscopic tools. The antimicrobial assessment of the target compounds **5a**–**n** was performed against a panel of Gram-positive and -negative bacteria as well as filamentous and non-filamentous fungi using both DIZ and MIC assays. Compound **5c** was the most active congener toward most of the tested Gram-positive isolates, while compound **5i** was the most active candidate against the Gram-negative isolate, *Ps. aeruginosa*. Compounds **5g** and **5h** were equipotent toward *P. notatum*, with an MIC value of 7.8 µg/mL, making it approximately 32 times more potent than FLC (MIC value of 250 µg/mL). Compound **5j** showed the best anti-*C*. *albicans* activity, with an MIC value of 3.9 µg/mL, making it about 4 times more potent than the antifungal reference standard FLC. The antifungal profile of the title compounds **5a**–**n** seemed to be better than their antibacterial profile. It is believed that the results of the current study could aid the development of new bioactive isatin-based antimicrobial molecules to be harnessed in clinics.

## Figures and Tables

**Table 1:** Diameter of the inhibition zone (DIZ) of the title compounds **5a**–**n**, ampicillin (AMP) and fluconazole (FLC) toward Gram-negative and -positive bacteria and fungi.

Comp. No.	DIZ in mm ± S.D. *											
	Strain											
	Gram-Negative Bacteria					Gram-Positive Bacteria				Fungi		
	*E. coli*	*Ps.aeruginosa*	*P. vulgaris*	*K. pneumonia*	*S. enteridis*	*S. aureus*	MRSA	*E. fecalis*	*B. subtilis*	*C. albicans*	*A. niger*	*P. notatum*
**5a**	14 ± 0.6	8 ± 0.8	−ve	−ve	−ve	14 ± 0.8	−ve	−ve	−ve	−ve	−ve	11 ± 0.3
**5b**	10 ± 1.0	8 ± 0.4	−ve	−ve	−ve	18 ± 0.4	18 ± 0.5	−ve	18 ± 1.2	12 ± 0.7	−ve	13 ± 0.0
**5c**	12 ± 2.0	8 ± 0.4	−ve	−ve	−ve	18 ± 0.4	18 ± 0.0	−ve	12 ± 1.6	10 ± 0.0	−ve	−ve
**5d**	10 ± 0.5	10 ± 0.0	−ve	−ve	−ve	12 ± 0.0	−ve	−ve	11 ± 0.9	12 ± 0.0	−ve	−ve
**5e**	−ve	9 ± 0.6	−ve	−ve	9 ± 0.3	9 ± 0.0	9 ± 0.0	9 ± 0.2	13 ± 0.4	20 ± 0.2	18 ± 0.4	15 ± 0.9
**5f**	10 ± 0.8	10 ± 1.0	−ve	12 ± 1.9	−ve	20 ± 1.0	14 ± 0.7	−ve	14 ± 0.6	10 ± 0.0	11 ± 0.0	9 ± 0.6
**5g**	20 ± 0.9	14 ± 1.8	12 ± 0.7	−ve	10 ± 0.4	22 ± 0.43	−ve	12 ± 0.0	12 ± 0.0	18 ± 0.0	16 ± 0.5	13 ± 0.0
**5h**	12 ± 1.2	18 ± 0.4	−ve	12 ± 1.76	−ve	18 ± 0.4	12 ± 0.3	−ve	12 ± 0.0	18 ± 0.5	11 ± 0.0	11 ± 0.12
**5i**	10 ± 1.4	16 ± 0.9	−ve	12 ± 1.6	−ve	18 ± 0.9	14 ± 0.6	−ve	12 ± 1.0	14 ± 0.7	−ve	−ve
**5j**	−ve	11 ± 0.4	−ve	−ve	9 ± 0.1	15 ± 0.6	9 ± 0.0	9 ± 0.5	18 ± 0.8	29 ± 0.0	15 ± 1.1	14 ± 0.2
**5k**	−ve	9 ± 0.0	−ve	−ve	11 ± 0.0	15 ± 0.0	9 ± 0.6	9 ± 0.0	16 ± 0.12	18 ± 1.0	18 ± 0.0	−ve
**5l**	−ve	9 ± 0.0	−ve	−ve	11 ± 0.1	21 ± 0.5	9 ± 0.2	14 ± 0.5	11 ± 0.8	16 ± 0.0	8 ± 0.0	−ve
**5m**	−ve	9 ± 0.0	−ve	−ve	11 ± 0.0	18 ± 1.1	9 ± 0.0	9 ± 0.0	20 ± 0.6	18 ± 0.0	14 ± 0.0	−ve
**5n**	−ve	12 ± 0.4	−ve	−ve	11 ± 0.3	18 ± 0.4	16 ± 0.6	9 ± 0.6	16 ± 1.0	18 ± 0.0	18 ± 0.0	−ve
**AMP**	30 ± 0.0	−ve	36 ± 0.7	−ve	45 ± 1.0	32 ± 0.4	18 ± 0.4	35 ± 1.0	30 ± 0.5	ND	ND	ND
**FLC**	ND	ND	ND	ND	ND	ND	ND	ND	ND	21 ± 0.5	16 ± 0.8	15 ± 0.0

* Arithmetic mean ± standard deviation; −ve: 0.0 mm; ND: not determined.

**Table 2:** Minimum inhibitory concentrations (MICs) of the title compounds **5a**–**n**, ampicillin (AMP) and fluconazole (FLC) toward Gram-negative and -positive bacteria and fungi.

MIC Values (µg/mL)												
Comp. No.	Strain											
	Gram-Negative Bacteria					Gram-Positive Bacteria				Fungi		
	*E. coli*	*Ps. aeruginosa*	*P. vulgaris*	*K. pneumonia*	*S. enteridis*	*S. aureus*	MRSA	*E. fecalis*	*B. subtilis*	*C. albicans*	*A. niger*	*P. notatum*
**5a**	250	500	500	500	500	1000	1000	1000	250	125	500	125
**5b**	250	500	500	500	500	15.6	31.25	125	15.6	7.8	>1000	31.25
**5c**	500	500	500	500	500	15.6	15.6	62.5	15.6	31.25	>1000	31.25
**5d**	250	500	500	500	500	1000	500	500	500	7.8	500	62.5
**5e**	250	125	250	500	500	1000	1000	500	500	15.6	15.6	62.5
**5f**	250	500	500	500	500	>1000	>1000	500	125	31.25	31.25	15.6
**5g**	500	125	125	500	1000	500	500	500	250	7.8	15.6	7.8
**5h**	250	500	250	500	500	500	500	>1000	250	31.25	31.25	7.8
**5i**	250	62.5	125	1000	500	500	500	>1000	250	62.5	62.5	31.25
**5j**	250	250	250	500	1000	500	>1000	1000	250	3.9	31.25	62.5
**5k**	250	250	1000	500	500	250	500	250	250	62.5	62.5	125
**5l**	250	250	1000	500	>1000	62.5	500	125	250	125	250	250
**5m**	250	500	1000	1000	500	125	500	250	500	62.5	125	250
**5n**	250	250	250	500	500	250	1000	250	250	62.5	62.5	250
**AMP**	15.6	>1000	<7.8	>1000	<7.8	250	500	3.9	1000	ND	ND	ND
**FLC**	ND	ND	ND	ND	ND	ND	ND	ND	ND	15.6	31.25	250

ND: not determined.
